# A Comparison of Reimbursement Recommendations by European HTA Agencies: Is There Opportunity for Further Alignment?

**DOI:** 10.3389/fphar.2017.00384

**Published:** 2017-06-30

**Authors:** Nicola Allen, Lawrence Liberti, Stuart R. Walker, Sam Salek

**Affiliations:** ^1^Centre for Innovation in Regulatory ScienceLondon, United Kingdom; ^2^School of Pharmacy and Pharmaceutical Sciences, Cardiff UniversityCardiff, United Kingdom; ^3^Department of Pharmacy, School of Life and Medical Sciences, Pharmacology and Postgraduate Medicine, University of HertfordshireHatfield, United Kingdom; ^4^Health Economic and Outcome Research, Institute for Medicines DevelopmentCardiff, United Kingdom

**Keywords:** new active substances, health technology assessment, HTA, process taxonomy, system taxonomy, reimbursement recommendations, archetypes, pharmaceuticals

## Abstract

**Introduction:** In Europe and beyond, the rising costs of healthcare and limited healthcare resources have resulted in the implementation of health technology assessment (HTA) to inform health policy and reimbursement decision-making. European legislation has provided a harmonized route for the regulatory process with the European Medicines Agency, but reimbursement decision-making still remains the responsibility of each country. There is a recognized need to move toward a more objective and collaborative reimbursement environment for new medicines in Europe. Therefore, the aim of this study was to objectively assess and compare the national reimbursement recommendations of 9 European jurisdictions following European Medicines Agency (EMA) recommendation for centralized marketing authorization.

**Methods:** Using publicly available data and newly developed classification tools, this study appraised 9 European reimbursement systems by assessing HTA processes and the relationship between the regulatory, HTA and decision-making organizations. Each national HTA agency was classified according to two novel taxonomies. The System taxonomy, focuses on the position of the HTA agency within the national reimbursement system according to the relationship between the regulator, the HTA-performing agency, and the reimbursement decision-making coverage body. The HTA Process taxonomy distinguishes between the individual HTA agency's approach to economic and therapeutic evaluation and the inclusion of an independent appraisal step. The taxonomic groups were subsequently compared with national HTA recommendations.

**Results:** This study identified European national reimbursement recommendations for 102 new active substances (NASs) approved by the EMA from 2008 to 2012. These reimbursement recommendations were compared using a novel classification tool and identified alignment between the organizational structure of reimbursement systems (System taxonomy) and HTA recommendations. However, there was less alignment between the HTA processes and recommendations.

**Conclusions:** In order to move forward to a more harmonized HTA environment within Europe, it is first necessary to understand the variation in HTA practices within Europe. This study has identified alignment between HTA recommendations and the System taxonomy and one of the major implications of this study is that such alignment could support a more collaborative HTA environment in Europe.

## Introduction

The rising cost of healthcare in the developed world, limited healthcare resources of individual jurisdictions and the need to improve quality and consistency of care, have resulted in the implementation of health technology assessment (HTA) to inform health policy and reimbursement decision making. Generally, HTA evaluates the added therapeutic benefits and risks for covering a health technology in the context of local standard of care (Allen et al., 2013). In Europe, European legislation has harmonized the regulatory process with the European Medicines Agency now responsible for granting marketing authorization, but reimbursement decision-making remains the responsibility of each country (European Commission, 2004; Allen et al., 2013). Patients also have access to a wealth of knowledge and increased opportunities for communication and collaboration. This increases expectations of and demands on the healthcare system and more informed patients are now aware of inequalities in patient access to new medicines in different jurisdictions (Beyer et al., 2007). However, there are also similarities between national approaches for HTA and this could provide opportunities for a more aligned, objective, and collaborative HTA environment in Europe (Henshall, 2012; Allen et al., 2013).

The European Network for Health Technology Assessment (EUnetHTA) was established in 2006 to create a sustainable European network for HTA and to develop and implement tools to transfer information between members (Kristensen, 2012). EUnetHTA was based on previous collaborative projects such as EUR-ASSESS and The European Collaboration for Health Technology Assessment (Velasco-Garrido et al., 2008). EUnetHTA was initially granted 3 years funding from the European Commission and the European Commission has since supported the formation of a permanent European HTA network with the scientific and technical cooperation of the new HTA Network conducted by EUnetHTA through Joint Action 3 until 2020 (Kristensen, 2012; European Commission, 2013; European Network for Health Technology Assessment (EUnetHTA), 2016). This continues EUnetHTA's support of Article 15 of the Directive for cross-border healthcare that requires “that the Union shall support and facilitate cooperation between national authorities or bodies responsible for HTA” (European Network for Health Technology Assessment (EUnetHTA), 2016). Meanwhile, the European Commission has initiated a process to determine support for European HTA beyond 2020 (European Commission, 2016a).

**Figure 1:**
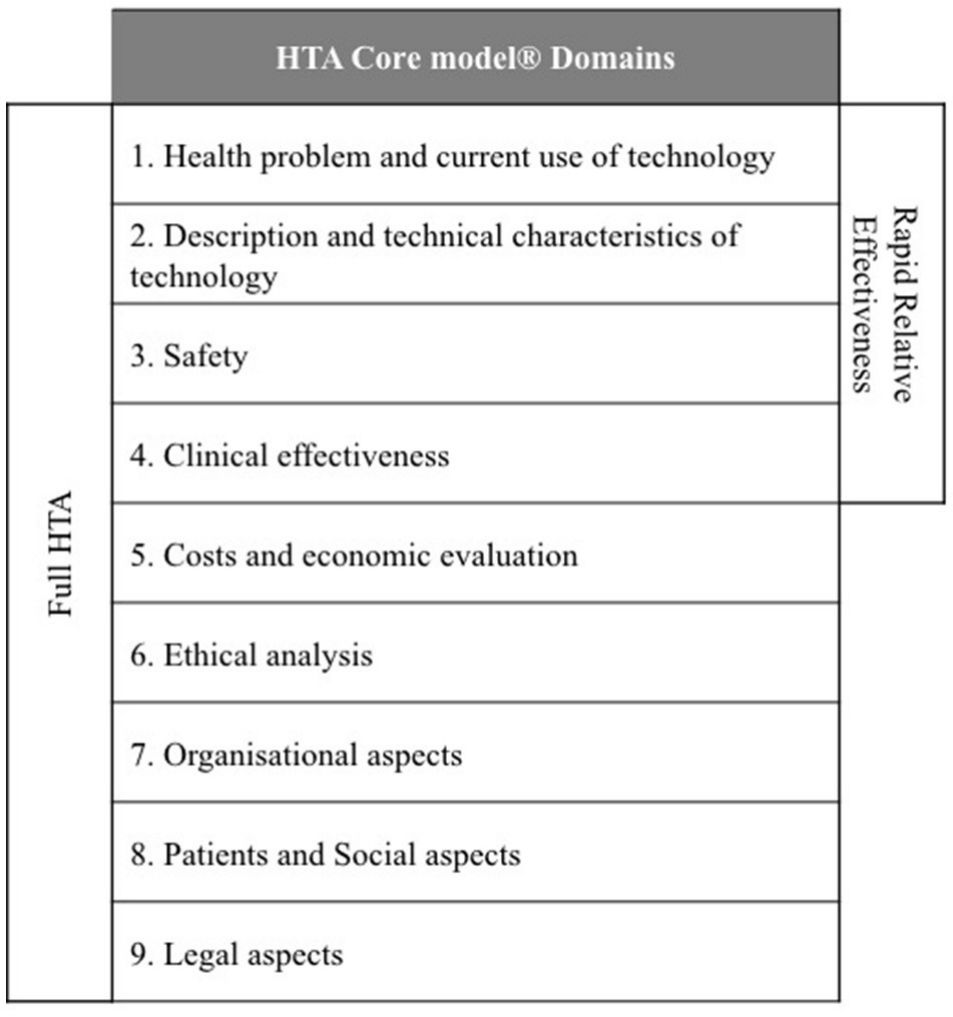
EUnetHTA HTA Core model® Domains.

According to Kristensen, one of the most innovative scientific and practical outputs of EUnetHTA has been the development of a HTA Core Model® that defines the key content required for HTA to support information sharing between HTA agencies (Kristensen, 2012). The HTA Core Model® contains 9 domains and each provide a framework for analysis (National Institute for Health and Welfare, 2014): description and technical characteristics of technology; health problem and current use of technology; safety; clinical effectiveness; cost and economic evaluation; ethical aspects; organizational aspects; social aspects; and legal aspects (Figure 1). The HTA Core Model® was designed to enable the sharing of HTA information in a common format that can be transferred between members at the national and international level and the 9 domains can be split into two groups: technical (health problem, description of technology, safety, and clinical effectiveness) and other (economic etc.) (Figure 1).

Pilots of the EUnetHTA HTA Core Model® for the rapid Relative Effectiveness Assessment (REA) focus on the technical domains (Charles River Associates, 2015). Ascroft and Pichler questioned the practicalities of the pilots, which resulted in the inclusion of local and regulatory data that was considered to be outside of the scope of the rapid REA (Ascroft and Pichler, 2014). This raised concerns that the application of the rapid REA could introduce a duplication of effort and questioned whether EUnetHTA members are likely to have the capacity to extend the pilot to review all the medicines and technologies that are reviewed by the EMA. Ascroft and Pichler (2014) have also proposed that the rapid REA could provide value by developing a REA report to supplement the current European Public Assessment Reports (EPARs).

The European Commission, acknowledging the potential benefits of increased European collaboration for HTA, has recently conducted a stakeholder consultation and is undertaking an inception impact assessment for strengthening of the EU cooperation on HTA (European Commission, 2016a). The inception impact assessment will consider various future scenarios for EU support of HTA including: continuing current activities until 2020; long-term voluntary cooperation funded by the EU beyond 2020; cooperation on collecting, sharing, and use of common data and tools; cooperation on joint REA reports or full HTA reports and their uptake (European Commission, 2016b). The potential for the establishment of a pan-European HTA agency has previously been explored by Drummond (2003), but this will require harmonization of guidelines, decision-making and willingness to pay.

The need for a more collaborative HTA environment has also been acknowledged beyond Europe. Prior to 2002, Canadian provinces and territories conducted their own assessments of the added therapeutic benefit and cost-effectiveness of NASs (Allen et al., 2016). The Canadian Agency for Drugs and Technologies in Health (CADTH), Common Drug Review (CDR) was implemented in 2002 to help standardize drug coverage across Canada by maximizing the use of resources and avoiding the duplication of work (Allen et al., 2016).

The need for a more collaborative HTA environment is also acknowledged at a global level. The Sixty-seventh World Health Assembly acknowledged the importance of regional and international collaboration on HTA and “urges Member States:…to consider also collaborating with other Member States' health organizations, academic institutions, professional associations and other key stakeholders in the country or region in order to collect and share information and lessons” (WHO, 2014).

There is also a recognized need to objectively describe and classify HTA systems and two novel non-ranking taxonomies for the classification of HTA performing agencies have been developed and described by Allen and colleagues (Henshall, 2012; Allen et al., 2013). This study utilized these classification methods to evaluate and compare HTA systems and processes for reimbursement recommendations for 9 European jurisdictions following EMA approvals for new active substances (NASs). In a previous study, the combination of two taxonomic sets, were used to identify different archetype groups within Europe with a view to identifying potential groupings for collaboration (Allen et al., 2013). The two aims of this study were to: (1) examine the relationship between the HTA System and Process taxonomies and the HTA recommendations for NASs and (2) to consider whether there is opportunity for further alignment.

## Methods

The EMA online database was searched for NASs granted marketing authorization between January 1, 2008 and December 31, 2012. For the purpose of this study, an *NAS* was defined as a chemical, biological, biotechnology, or radiopharmaceutical substance that has not been previously available in Europe for therapeutic use in humans and is destined to be made available as a prescription only medicine for humans. Generics, vaccines, and products previously licensed for sale in any European jurisdiction were excluded from this study and only publicly available data were used.

The initial HTA recommendations for each NAS and its indications reviewed for reimbursement were identified from the official national agency websites for 9 jurisdictions: Belgium, England, France, Germany, Ireland, Italy, Netherlands, Scotland, and Sweden. These 9 jurisdictions were selected for inclusion in this study as they publish information on their reimbursement assessments in the public domain and they cover a range of taxonomies as identified in previous research (Allen et al., 2013).

**Figure 2:**
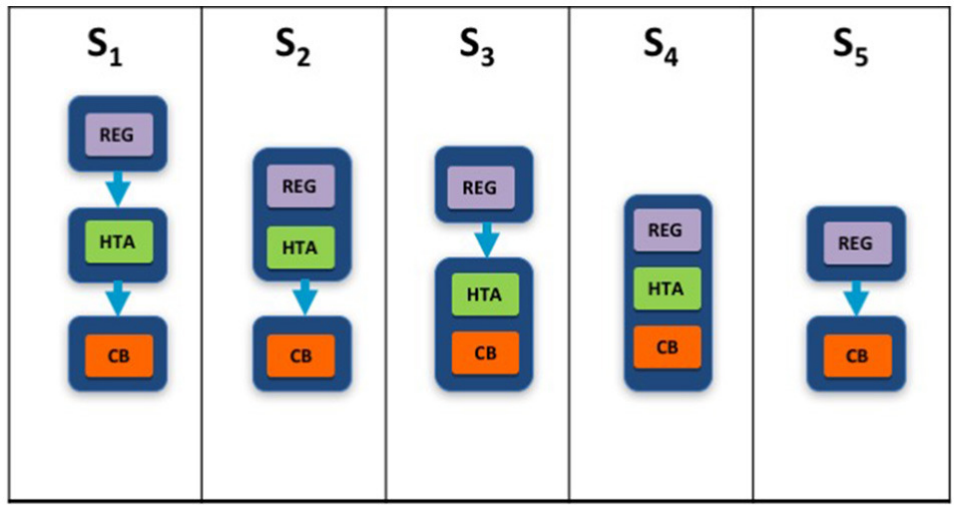
The System taxonomy includes five subsets and is based on the position of three core functions: a national HTA performing agency, if present, in relation to the position of the regulatory authority (REG) and the decision-making coverage body (CB). The five-subset of the System taxonomy: S_1_–the regulatory, HTA and coverage body functions are performed by separate agencies. S_2_–the regulatory and HTA functions are performed by a single agency and the coverage body functions are independent. S_3_–the HTA and coverage body functions are performed by a single agency with the regulatory function performed independently. S_4_–the regulatory, HTA and coverage body functions are all performed within a single agency. S_5_–no HTA is performed within the national regulatory to reimbursement system.

Two taxonomic sets were developed by comparing HTA process maps for 33 European jurisdictions to demonstrate the communication and information flow pathways between the sponsor (manufacturer) and key agencies within the national reimbursement system (Allen et al., 2013). The System taxonomy, focuses on the position of the HTA agency within the national reimbursement system according to the relationship between the regulator, the HTA-performing agency, and the reimbursement decision-making coverage body. Five-subsets are included within the System taxonomy (Figure 2):

S_1_–The regulatory, HTA and coverage body functions are performed by separate agencies
S_2_–The regulatory and HTA functions are performed by a single agency and the coverage body functions are independent
S_3_–The HTA and coverage body functions are performed by a single agency with the regulatory function performed independently
S_4_–The regulatory, HTA and coverage body functions are all performed within a single agency
S_5_–No HTA is performed within the national regulatory to reimbursement system

The interactions between the three core functions of the System taxonomy can ultimately affect overall system performance. Drummond et al. (2008) have stressed the importance of conducting HTA independently of the decision-making body to reduce bias, and this System taxonomy permits the classification of jurisdictions based on the independence of the HTA process.

**Figure 3:**
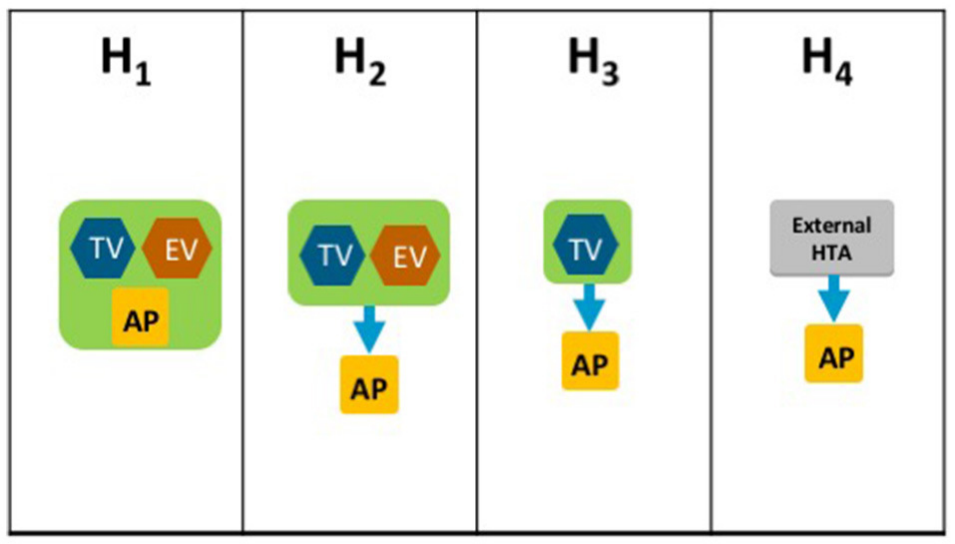
The HTA process taxonomy includes four subsets and focuses on the key tasks performed by the HTA agency. Each group shows the relative positions of three key tasks, if performed, within the HTA agency: therapeutic value (TV), economic value (EV), and appraisal (AP). The four-subsets of the HTA Process taxonomy: H_1_–the therapeutic value assessment, economic evaluation and appraisal are performed within the same agency. H_2_–the therapeutic value assessment is conducted within the same agency as Economic evaluation but the appraisal is performed independently, usually by health professionals rather than civil servants. H_3_–the therapeutic value is assessed prior to independent appraisal. H_4_–the appraisal is conducted using information from an external HTA report or by considering the coverage decisions of reference countries.

The HTA Process taxonomy distinguishes between the individual HTA agency's approach to economic and therapeutic evaluation and the inclusion of an independent appraisal step. Four-subsets were identified for the HTA Process taxonomy (Figure 3):

H_1_–The therapeutic value assessment, economic evaluation and appraisal are performed within the same agency
H_2_–The therapeutic value assessment is conducted within the same agency as Economic evaluation but the appraisal is performed independently, usually by health professionals rather than civil servants
H_3_–The therapeutic value is assessed prior to independent appraisal
H_4_–The appraisal is conducted using information from an external HTA report or by considering the coverage decisions of reference countries.

The inclusion of an independent appraisal step can reduce the perception of bias and is therefore an important factor that should be considered for the purpose of classification (Drummond et al., 2008). However, not all agencies adopt this approach for medicines and may conduct their own assessment as it is the manufacturer's dossier that is submitted for appraisal.

Each HTA recommendation or reimbursement decision was categorized as, *recommended, recommended with restrictions*, or *not recommended*. These three HTA recommendation categories were subsequently numerically coded for direct comparison between agency pairs to enable identification of the total number of aligned recommendations. For each pair of agencies (jurisdictional pairs), the total number of medicine—indication pairs reviewed by both agencies were identified and the proportion of congruent recommendations were calculated by percentage agreement. The percentage agreements for jurisdictional pairs that were allocated to the same taxonomic subsets were compared with the percentage agreements for jurisdictional pairs from different subsets to identify potential alignment.

## Results

### Comparison of HTA recommendations by 9 european HTA agencies

**Figure 4:**
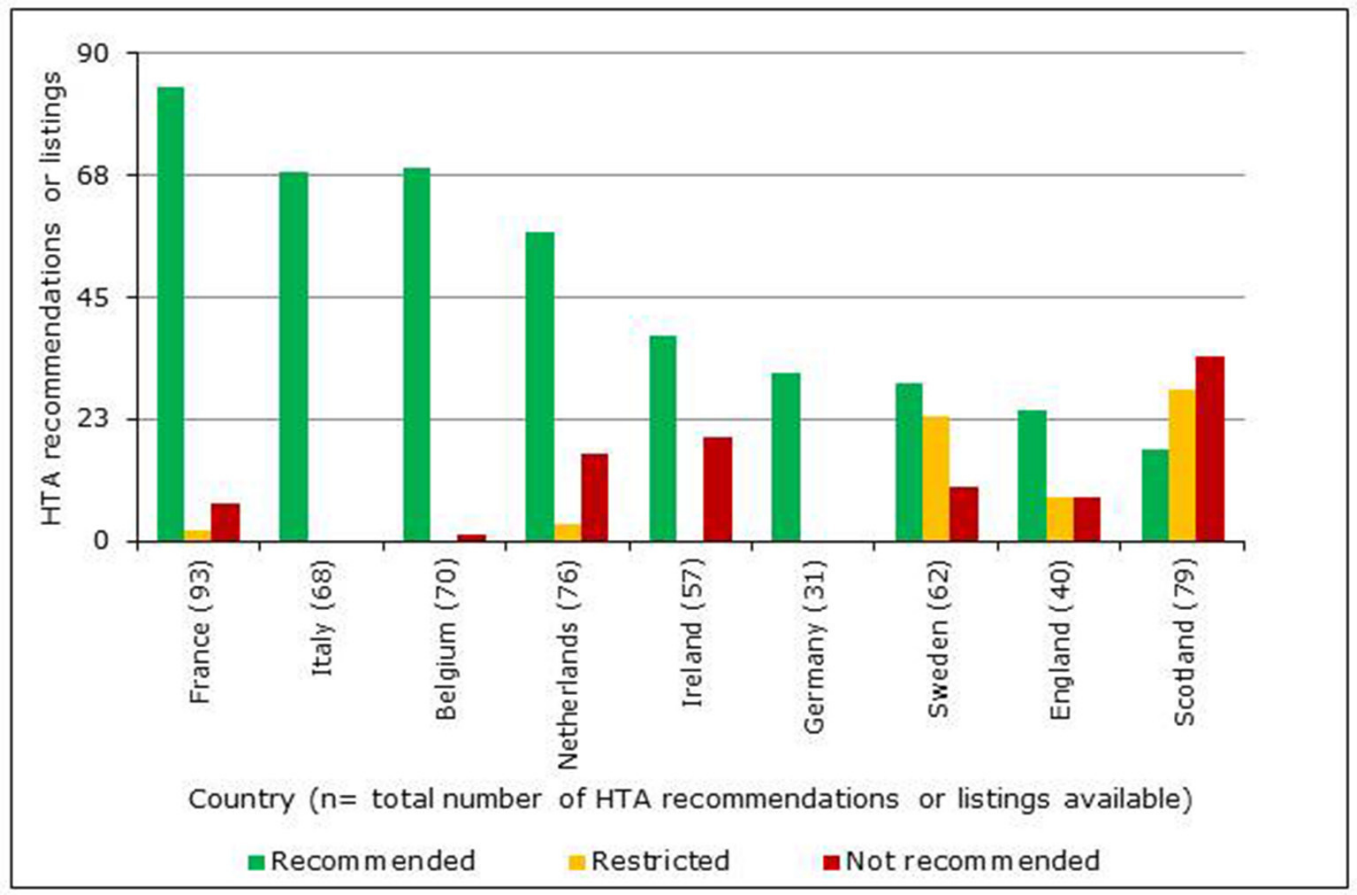
Number of HTA reimbursement recommendations for 9 European jurisdictions classified according to recommendation category.

A total of 102 NASs received a central marketing authorization in accordance with regulation 726/2004 between January 1, 2008 and December 31, 2012 (European Commission, 2004). However, the reimbursement information available for these NASs varied between agencies, as only data available in the public domain by August 2014 were used (Table 1). The HTA reimbursement recommendations for the 9 European jurisdictions included in the study are shown in Figure 4. Reimbursement recommendations for England were available for 39% of the NASs included in this study, as the National Institute for Health and Care Excellence (NICE) only conduct appraisals for NASs expected to have a “significant impact” (National Institute for Health and Care Excellence (NICE), 2017). Medicines that are not reviewed by NICE can be considered for reimbursement through NHS England or Clinical Commissioning Groups. The publically available recommendations from the German Federal Joint Committee (G-BA) early benefit assessment are only available from January 1, 2011, which was reflected in their low number of recommendations. In contrast with the other jurisdictions, all recommendations issued by the G-BA have been allocated to the universal reimbursed category; this difference occurs because the G-BA recommendation only gives a score for the added therapeutic benefit to inform price negotiations. Reimbursement recommendations from The French National Authority for Health (HAS) also provide a score for added therapeutic benefit, but recommendations can also include restrictions or a rejection of the application.

**Table 1:** Reimbursement recommendations of 9 European Union jurisdictions for NASs receiving EMA marketing authorization in accordance with regulation 726/2004 between January 1, 2008 and December 31, 2012.

**Jurisdiction (Agency)**	**Reimbursed**	**Reimbursed with restrictions**	**Not reimbursed**	**Proportion of medicines receiving marketing authorization to receive a recommendation at time of study (%)**	**Website**
Belgium (Belgium Health Insurance Agency; INAMI)	Insured (Class 1)	N/A	Not reimbursed	69	www.inami.be
	Insured (Class 2)				
England (National Institute for Health and Care Excellence; NICE)	Recommended	Optimized	Not recommended	39	www.nice.org.uk
France (French National Authority for Health; HAS)	Approved	Approved with restriction	Not recommended	91	www.has-sante.fr
Germany (Federal Joint Committee; G-BA)	Indication of a considerable additional benefit			30	www.english.g-ba.de
	Hint of considerable additional benefit				
	Proof of a significant additional benefit				
	Minor additional benefit				
	Additional benefit has not been proved				
Ireland (National Centre for Pharmacoeconomics; NCPE)	Reimbursement recommended		Reimbursement not recommended	56	www.ncpe.ie
			Reimbursement not recommended at submitted price		
Italy (Italian Medicines Agency; AIFA)	Reimbursed Class A			67	www.agenziafarmaco.gov.it
	Reimbursed class H				
Netherlands (National Health Care Institute; ZiNL)	Insured (Annex 1A)	Insured with restrictions	Not recommended	75	www.zorginstituutnederland.nl
	Insured (Annex 1B)				
Scotland (Scottish Medicines Consortium; SMC)	Accepted	Restricted	Not recommended	77	www.scottishmedicines.org.uk
Sweden (Dental and Pharmaceutical Benefits Agency; TLV)	General	Limitations	Not recommended	61	www.tlv.se

In Ireland, no outcome was recorded when there was only a recommendation to conduct a full pharmacoeconomic review. In addition, the assessment information available in Ireland did not include a category that could be classified as *restricted*. In Italy, NASs are reviewed by the Italian Medicines Agency for inclusion in their national formulary. This formulary includes three lists; *List A* for fully reimbursed products; *List H* for products only reimbursed in hospitals and *List C* for products not reimbursed (Italian Medicines Agency (AIFA), 2015). Only two of these lists were available online at the time of this study and neither list included approved indications or criteria for prescribing. Due to the ambiguity of these recommendations, Italy has been excluded from the final comparisons. Also excluded from the comparisons were the negative recommendations of the Scottish Medicines Consortium (SMC), which are made when sponsors (manufacturers) of a NAS fail to submit a dossier for review (Scottish Medicines Consortium (SMC), 2012). Positive recommendations for reimbursement by the Netherlands National Health Care Institute (ZiNL) can be categorized into two groups: *Annex 1a* for NASs that have a similar therapeutic value and are interchangeable and *Annex 1b* for NASs that have added therapeutic value.

The reimbursement decision pathways for NASs vary across the 9 jurisdictions compared in this study (including Italy) (Allen et al., 2013). They all involve input from the sponsor and communication between the market authorities: the regulator, the pricing authorities, the recommender, and the decision maker within each jurisdiction. For example, NICE conducts horizon scanning and receives requests from the Department of Health to identify potential NASs for review.

### Comparison of HTA recommendations and taxonomic classifications

A total of 28 unique jurisdictional pair combinations and their congruence (percentage agreement) were evaluated and displayed in a cross tabulation format (Tables 2, 3). These 28 pairs present the proportion of congruent recommendations for the total number of medicines reviewed by both jurisdictions. The congruence percentages have been classified into three grades: high congruence (≥75%); medium congruence (≥ 50% to 74%); and low congruence (<50%). In each table, the jurisdictions were grouped according to their respective subsets within each of the two taxonomies (Figures 2, 3). For the System Taxonomy, the S1 set includes Germany, Ireland, France, Netherlands, and Belgium and the S3 set includes England, Scotland, and Sweden. None of the jurisdictions reviewed were classified as S2, S4, or S5 (Table 2). For the HTA process taxonomy, the H_1_ subset includes Scotland, Ireland, and Sweden; the H_2_ set includes England and Belgium and the H_3_ subset includes Germany, France, and the Netherlands. None of the jurisdictions reviewed were classified as H_4_ taxonomy (Table 3).

**Table 2:**
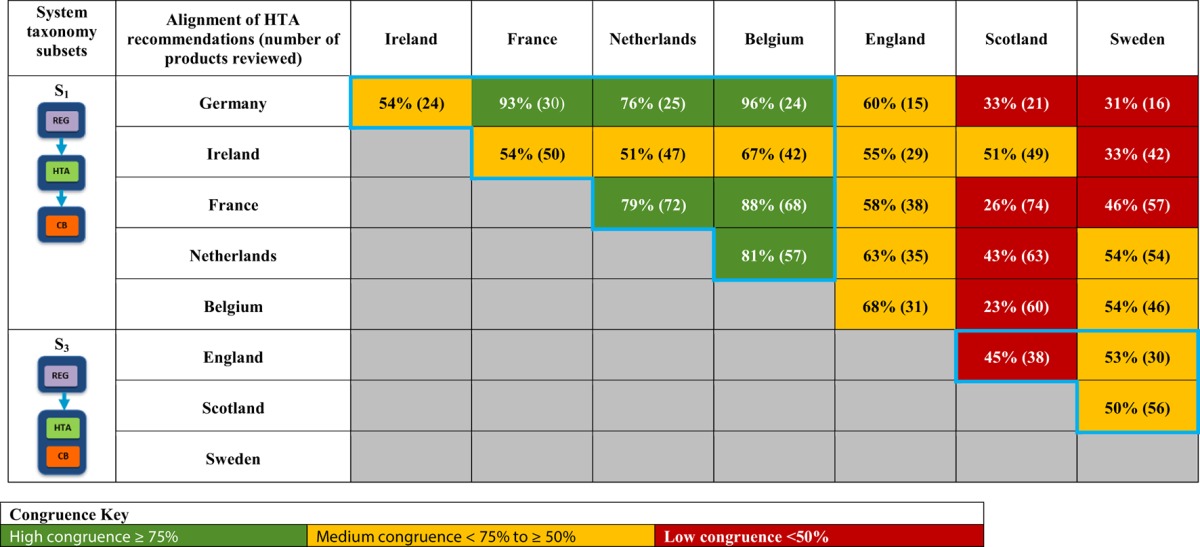
Alignment of HTA recommendations and reimbursement decisions allocated to three categories by the System taxonomy.

*For the System Taxonomy sets outlined, the S_1_ set includes Germany, Ireland, France, Netherlands, and Belgium and the S_3_ set includes England, Scotland, and Sweden. None of the jurisdictions reviewed were classified as S_2_, S_4_, or S_5_*.

**Table 3:**
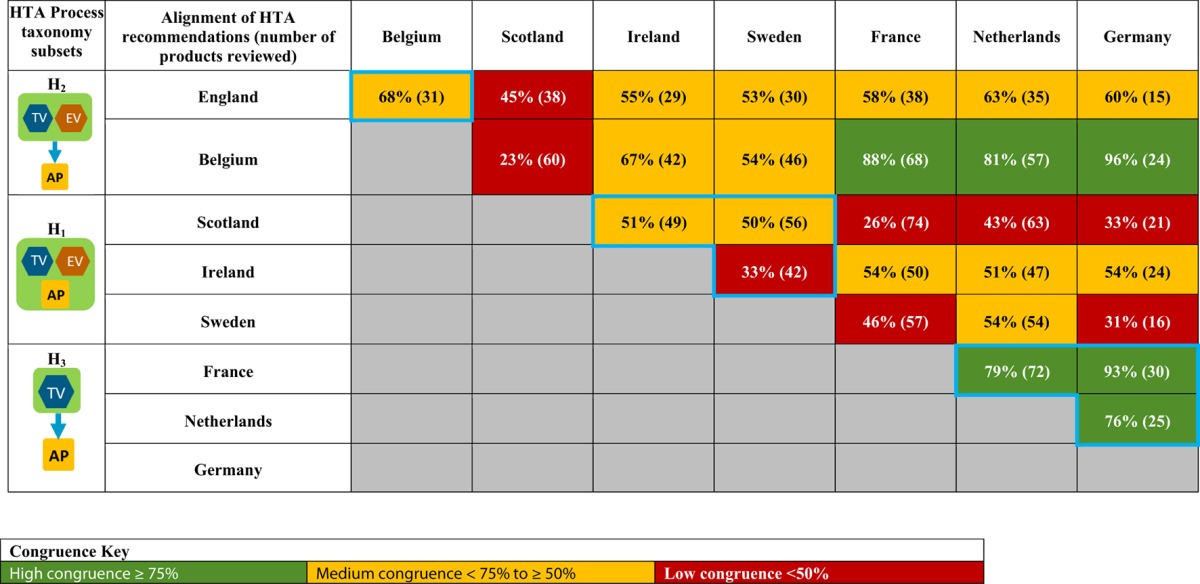
Alignment of HTA recommendations and reimbursement decisions allocated to three categories by the HTA Process taxonomy.

*For the HTA process taxonomy sets outlined, the H_1_ set includes Scotland, Ireland and Sweden; the H_2_ set includes England and Belgium and the H_3_ set includes Germany, France and the Netherlands. None of the jurisdictions reviewed were classified as H_4_ taxonomy*.

**Figure 5:**
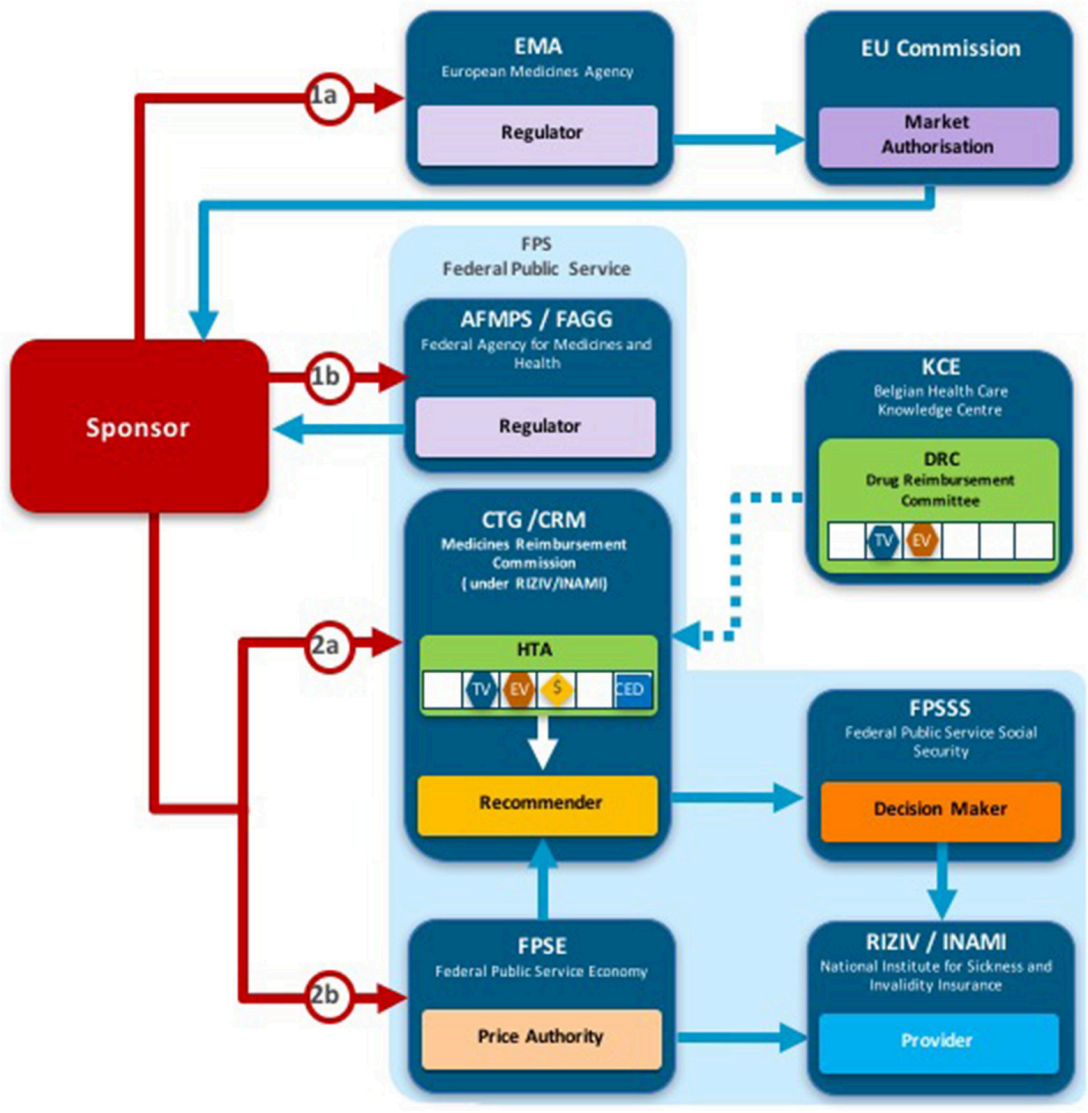
Process maps for Belgium.

**Figure 6:**
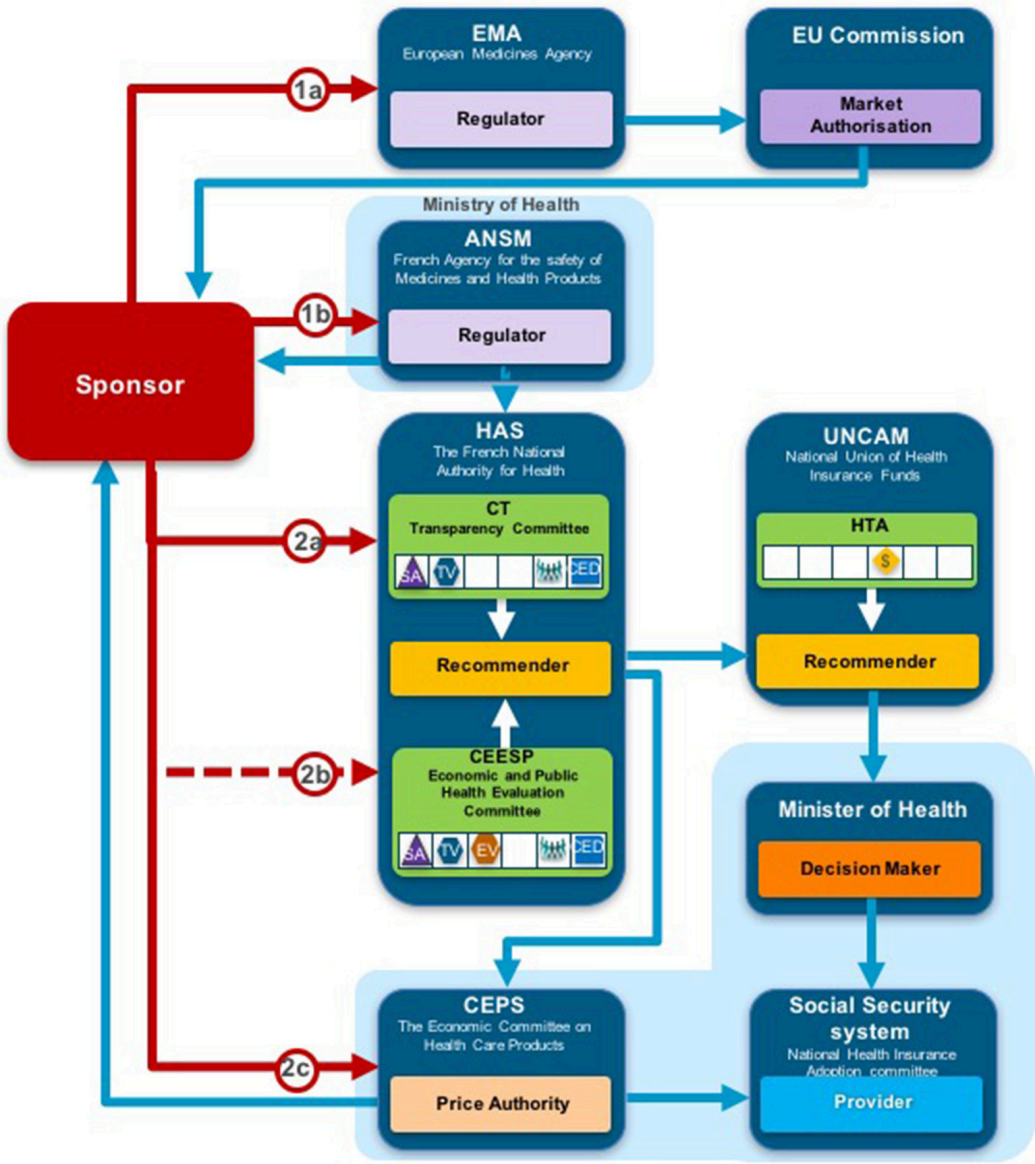
Process map for France.

**Figure 7:**
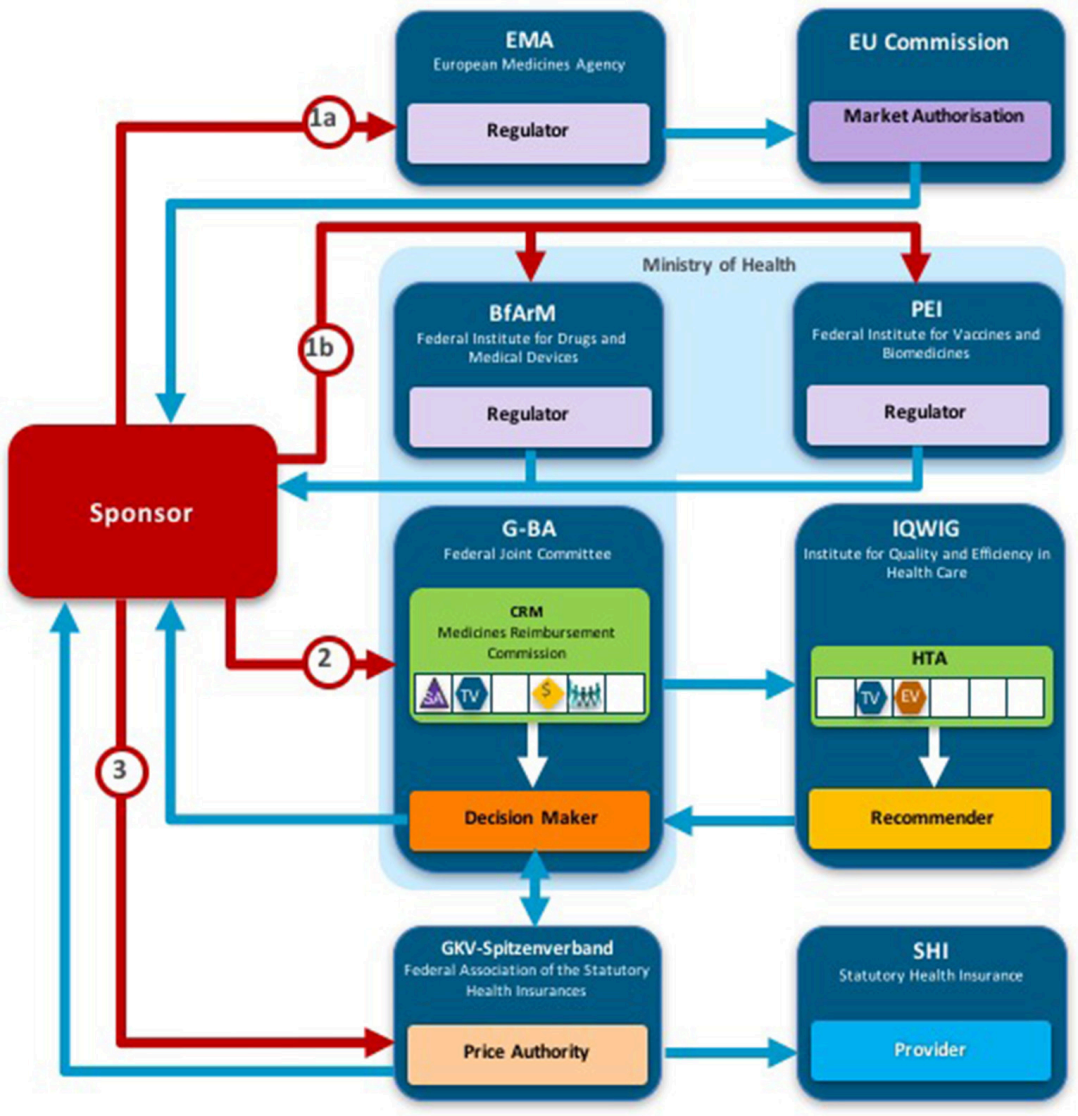
Process map for Germany.

**Figure 8:**
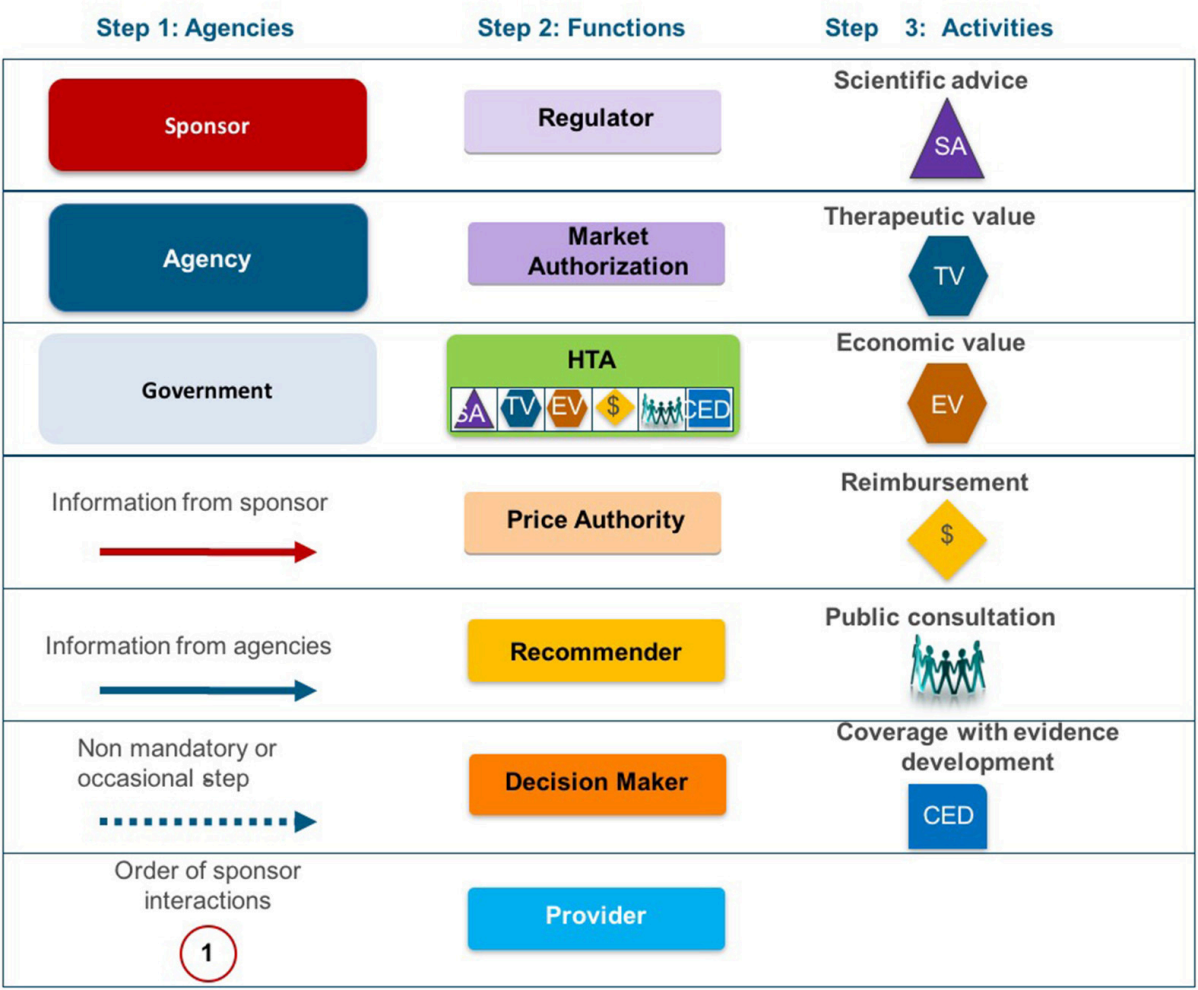
Process map key.

Overall, six of the country pair comparisons displayed high-level congruence (Belgium paired with Germany, France, or Netherlands, Germany paired with Netherlands or France and France paired with Netherlands). The strongest alignment (≥88%) was observed between Belgium, France, and Germany, which could be due to these agencies focus on therapeutic benefit (Figures 5–8). All six pairs are also located within the S_1_ taxonomic group, which is the largest group within the System taxonomy and represents a system that has separate agencies or organizations to perform the regulatory, HTA and decision-making functions (Table 2, Figure 2). All jurisdictional pairs within this S_1_ taxonomic group had a 50% or higher agreement for HTA recommendations and therefore scored either high or medium congruence. Taxonomic set S_3_ (England, Scotland, and Sweden) did not include any high congruence pairs and only included two medium and one low congruence pair.

These 28 jurisdictional pairs were arranged in a second cross tabulation in which jurisdictions were grouped according to their Process taxonomy (Table 3, Figure 3). The two jurisdictions in HTA process taxonomic group H_2_ (England, and Belgium) perform the therapeutic value and economic value assessment with an independent appraisal and scored medium congruence. Group H_1_ (Scotland, Ireland, and Sweden) scored two medium congruent pairs and one low congruent pair (Sweden and Ireland). Taxonomic group H3 included Germany, France and Netherlands with all three jurisdictional pairs presenting high congruence with a percentage agreement ranging from 76 to 93%. The distribution of high, medium-, and low-congruence pairs indicates alignment around the H_3_ subset, but does not indicate any clustering around the other HTA Process taxonomic groupings.

## Discussion

In order to move forward to a more harmonized HTA environment within Europe, it is first necessary to understand the variation in HTA practices there. The processes required for providing patients' access to new medicines has become more complex with the increased uptake of HTA to inform coverage decisions. Obtaining reimbursement is commonly referred to as the “fourth hurdle,” as this can require a positive HTA recommendation based on the relative clinical benefit and cost effectiveness of a new health technology following the regulatory assessment for safety, efficacy, and quality, while some also note a fifth hurdle: affordability. The European Union's establishment of the EMA successfully standardized the procedure for the review and approval of new medicines across Europe in accordance with regulation 726/2004 (European Commission, 2004). However, the pharmaceutical industry is still required to submit multiple applications to individual European countries for HTA and reimbursement assessments, which can result in varying reimbursement recommendations. Discrepancies between HTA recommendations may also be due to the quality of evidence available, willingness to accept uncertainty or differing methods of assessment or priorities (Allen et al., 2017). The methodologies and processes used to conduct HTA can vary from country to country and also between regions when decision making is decentralized (e.g., Italy and Spain). Thus, the pharmaceutical industry must learn to navigate an ever-changing patchwork of HTA agencies, as HTA methodologies and processes continue to evolve.

Manufacturers often submit applications first to markets where they are likely to achieve a higher price, as reasoned by Morton and Kyle (2012):

“…*a manufacturer should want to negotiate over prices and launch new products in high-price countries first, so as to positively affect any reference price used by later countries.”*

Many European countries will review prices achieved in other European markets to guide pricing. The regulation of drug prices varies between markets and this is taken into consideration when launching new medicines:

“…*the price on these markets is usually higher due to the lack of the regulation and it is therefore more profitable for the manufacturers to market the drugs in these countries first”* (Łanda et al., 2009).

This can result in patient access inequalities throughout Europe, as patients in countries that tend to pay higher prices are more likely to have earlier access, while patients in countries that are unable or not willing to provide coverage at a price obtained in other European countries may be denied access:

“*Studies concluded that pharmaceutical firms had incentives in launching new drugs in high-price countries first and delaying launch or even not launching new drugs in low-price countries”* (Toumi et al., 2014).

The time taken to prepare multiple submissions is also detrimental to the pharmaceutical industry as it reduces the time remaining with patent protection to recover research and development costs and generate a profit.

The results of this research highlight the varying approaches to HTA and the potential impact this can have on the reimbursement of new medicines. For example, the proportion of the 102 NAS Medicine-indication pairs reviewed by a single agency ranges from 30% (Germany) to 91% (France). The low proportion of recommendations reviewed by the German G-BA is due to the implementation of the new AMNOG requirements during the study period (Ruof et al., 2014). However, the 39% of medicines reviewed by NICE (England) is due to the agency's mandate to only review medicines of significant impact (National Institute for Health and Care Excellence (NICE), 2017). This is very different to other agencies that require a HTA for all new medicines, such as France and Scotland. The proportion of medicines reviewed is also dependent on the manufacturer's decision to submit a HTA dossier for review.

This study has evaluated the relationship between HTA agencies' recommendations, their classification into taxonomic sets and calculated the percentage agreement for all 28 possible combinations of jurisdictional pairs. The percentage agreement results demonstrate alignment between HTA recommendations and the System taxonomy. However, the results demonstrate less alignment between HTA recommendations and HTA Process taxonomic sets. This could be due to the diversity of the decision making processes and the heterogeneity of the systems compared, but may also suggest that further research is required to refine the archetypes for real-life application. It is also more difficult to allocate agencies to HTA Process taxonomic sets because it is not always clear how independent the clinical evaluation is from the economic evaluation. For example, the French National Authority for Health (HAS) now requires the submission of an economic dossier in parallel with the clinical submission, but only for NASs that have a high rating for improvement in medical benefit (ASMR I, II, or III) and an estimated total cost to the system of more than €20 million within 2 years of commercialization (Haute Autorité de Santé (HAS), 2015). Therefore, the HAS evaluation could be allocated to different HTA Process taxonomic sets, depending on the NAS evaluated.

### Comparison with similar studies

The comparisons of eight European HTA agencies in this study, build on previous research by Nicod and Kanavos (2012) and Bending et al. (2012). Nicod and Kanavos evaluated HTA recommendations from Australia, Canada, England, Scotland, and Sweden with a particular focus on therapeutic areas and identified significant variation between national recommendations. Bending et al. compared the processes and recommendations of two national HTA agencies (France and Scotland) to identify differences between agencies that include or exclude cost-effectiveness evaluations for reimbursement recommendations for new medicines. However, there are many factors that can cause discordant HTA recommendations and comparing only two agencies has limited value. Therefore, the comparisons of HTA recommendations from eight European HTA agencies are more likely to identify potential alignment of factors that impact reimbursement recommendations. The calculations for agreement between country pairs indicated that HTA agencies, classified by the System taxonomy, might correlate with concordant HTA recommendations. This is a novel result and a valuable outcome of the development of the taxonomies as a classification tool, with the implication from this study that such alignment could support a more collaborative HTA environment in Europe.

### Is there potential for a more aligned HTA environment in europe?

The establishment of the CDR in Canada has demonstrated a successful working model for sharing HTA evidence derived from a centralized review, to provide a more efficient use of resources to support regional decision making (Allen et al., 2016) Similarly, there is a general acceptance that decision making should remain at the national/local level in Europe, but this does not prevent collaborations for assessment (Kleijnen et al., 2015). The rationale underpinning the establishment of EUnetHTA has similarities with the rationale for the creation of the CDR, as both aim to reduce duplication of work, use HTA resources more efficiently, and provide access to robust scientific evidence. The disparities between medicines coverage across Canadian provinces was a key concern that led to the development of the CDR to provide a standard approach to HTA that could inform regional decisions (Allen et al., 2016). European countries are more heterogeneous than Canadian provinces and territories, but similar concerns have been raised regarding varying patient access to new medicines and EUnetHTA aims to support cross-border application of tools and methodologies for HTA (European Network for Health Technology Assessment (EUnetHTA), 2016).

Drummond (2003) argued that the creation of a European HTA agency is a possibility but three key challenges would need to be harmonized first: economic evaluation guidelines, decision-making processes, and societal willingness-to-pay for health technologies. He suggested that the harmonization of economic guidelines would be the easiest of the three challenges, but even with common European guidelines the differences between country policies may require tailored reports to enable inclusion or exclusion of data, such as productivity costs. Harmonizing societal willingness-to-pay is arguably the most challenging factor. Even if there were a single price for Europe, there would still be differences in the local costs for healthcare that may be required to deliver or monitor the medicine, which would affect the cost-effectiveness of the product. Overall, Drummond suggests the likelihood of all three achieving harmonization in the near future is very low.

Overall, there are two key aspects of the reimbursement decision that should be considered for evaluating the potential for harmonization: the technical evaluation and the final reimbursement decision. The safety and clinical effectiveness of a new medicine is a technical consideration that is relevant for all reimbursement decisions. However, the added therapeutic benefit of a new medicine is dependent on the chosen comparator and will impact the final reimbursement decision. Similarly, as proposed by Drummond (2003) HTA agencies that consider cost-effectiveness could align the economic guidelines, but the local costs considered and willingness to pay will be specific for each jurisdiction and thus difficult to harmonize the final decision. This study has evaluated the final reimbursement recommendation that considers more variables than the technical evaluation and identified alignement within certain taxonomic subsets. Therefore, one could assume that there could be greater alignment between the less variable technical evaluation and this is where initial collaboration efforts should focus. The European Commission's inception impact assessment on the strengthening of the EU cooperation on HTA will be exploring various options for European HTA collaboration at the end of 2017 (European Commission, 2016b).

A pan-European HTA agency is a controversial topic and it could be argued that it is not possible to harmonize HTA across Europe, as the countries are too different with respect to healthcare budgets and medical standards of care together with the political, social, and economic aspects of HTA, which are difficult to align. However, the Canadian HTA environment provides a working model for a centralized HTA agency that enables regions to include evidence generated at the national level that has been considered in the local context (Allen et al., 2016). It should also be noted that prior to the establishment of the EMA there were many who doubted the possibility of a single European regulatory authority, due to varying approaches across Europe. However, the EMA was established in 1995 and has now been successfully providing marketing authorization for medicines across Europe for more than 20 years.

### Limitations and future research

The two taxonomic sets have been used to compare HTA recommendations from eight European national agencies (excluding Italy). The number of HTA agencies included for comparison was limited due to the varied depth of information published online and the agencies included are fairly homogenous in regards to their economic development. Further, it was not possible to have a range of jurisdictional pairs for all taxonomic groupings, as HTA reimbursement recommendations were only compared for eight agencies. The HTA Process taxonomy contains at least two countries with national HTA recommendations for comparison (jurisdictional pairs) for all three of the HTA performing taxonomic sets; however, the “System taxonomy” does not contain any pairs for two of the four HTA performing taxonomic sets (S2 and S4) and this is a limitation for this study. Future studies could expand on this research by including at least two national HTA agencies for all HTA performing taxonomic sets and by comparing HTA recommendations over a longer period of time to identify trends. Comparing reimbursement recommendations across agencies with varying processes is challenging and this study grouped the different reimbursement recommendation options into three main groups (reimbursed, reimbursed with restrictions, and not reimbursed) to facilitate comparison. Not all agencies included in this study issue recommendations that fall within all three categories and this is a limitation to this research. The final reimbursement decision may also be influenced by the submitted price and whether an agency has a mandate to negotiate price or consider a managed entry agreement to share the financial risk. This was not a consideration in this study and future research could augment this study by evaluating the impact of price and managed entry agreements for HTA recommendations.

## Conclusion

This research has identified alignment between HTA recommendations and the System taxonomy, but less alignment was identified with the HTA process taxonomy. Therefore, it could be argued that there is a relationship between the regulatory, HTA, and decision-making functions in the healthcare system and the final HTA reimbursement recommendations, but further research would be needed to support this relationship. Understanding the disparities in HTA practices within Europe and the impact of these variations on patient access is necessary to move forward to a more harmonized HTA. Therefore, one of the major implications of this study is that such alignment could support a more collaborative HTA environment in Europe.

## Author contributions

NA, participated in the design of the study, acquired, and analyzed the data, developed the manuscript draft, and approved the final content. LL, SW, and SS participated in the design of the study and analysis of the data, helped to develop the manuscript draft and approved the final content.

### Conflict of interest statement

The authors declare that the research was conducted in the absence of any commercial or financial relationships that could be construed as a potential conflict of interest.

## Abbreviations

AIFA: Agenzia Italiana del Farmaco (Italian Medicines Agency)
CADTH: Canadian Agency for Drugs and Technologies in Health
CDR: Common Drug Review
EMA: European Medicines Agency
EPARS: European Public Assessment Reports
EUnetHTA: European Network for Health Technology Assessment
G-BA: Der Gemeinsame Bundesausschuss (German Federal Joint Committee)
HAS: Haute Autorité de santé (French National Authority for Health)
HTA: health technology assessment
INAMI: Institut National d'Assurance Maladie-Invalidité (Belgian National Institute for Health and Disability Insurance)
QWIG: Institut für Qualität und Wirtschaftlichkeit im Gesundheitswesen (German Institute for Quality and Efficiency in Healthcare)
NASs: new active substances
NCPR: National Centre for Pharmacoeconomics (Ireland)
NICE: National Institute for Health and Care Excellence (England)
REA: relative effectiveness assessment
SMC: Scottish Medicines Consortium
TLV: Tandvårds- & läkemedelsförmånsverket (Swedish Dental and Pharmaceutical Benefits Agency)
ZINL: Zorginstituut Nederland (Netherlands National Health Care Institute).
